# The Cognitive Costs of Context: The Effects of Concreteness and Immersiveness in Instructional Examples

**DOI:** 10.3389/fpsyg.2015.01876

**Published:** 2015-12-01

**Authors:** Samuel B. Day, Benjamin A. Motz, Robert L. Goldstone

**Affiliations:** ^1^Department of Psychology, Susquehanna University, Selinsgrove, PA, USA; ^2^Department of Psychological and Brain Sciences, Indiana University, Bloomington, Bloomington, IN, USA

**Keywords:** transfer, analogical transfer, analogical reasoning, learning, context, concreteness, cognition

## Abstract

Prior research has established that while the use of concrete, familiar examples can provide many important benefits for learning, it is also associated with some serious disadvantages, particularly in learners’ ability to recognize and transfer their knowledge to new analogous situations. However, it is not immediately clear whether this pattern would hold in real world educational contexts, in which the role of such examples in student engagement and ease of processing might be of enough importance to overshadow any potential negative impact. We conducted two experiments in which curriculum-relevant material was presented in natural classroom environments, first with college undergraduates and then with middle-school students. All students in each study received the same relevant content, but the degree of contextualization in these materials was varied between students. In both studies, we found that greater contextualization was associated with poorer transfer performance. We interpret these results as reflecting a greater degree of embeddedness for the knowledge acquired from richer, more concrete materials, such that the underlying principles are represented in a less abstract and generalizable form.

## Introduction

Educators at all levels are frequently encouraged to incorporate concrete, meaningful, real-world examples into their lessons (e.g., Rivet and Krajcik, 2008). In fact, this advice probably seems like a truism to most teachers. For instance, although the principles of Mendelian inheritance could be discussed in an entirely abstract and general way, instructors are far more likely to introduce these ideas in terms of specific, familiar, real-world domains such as eye color or the traits of pea plants. There are solid practical and theoretical reasons for couching new material in terms of familiar examples. Maintaining student attention and engagement is a constant concern, and research shows that more concrete materials tend to increase student interest (Sadoski et al., 1993). Furthermore, there is considerable evidence that familiar and concrete examples are simply easier to process than more abstract representations. For example, in the original abstract version of the Wason (1966) selection task (a classic test of conditional reasoning), only 4% of the participants were able to generate the logically correct solution. However, when the task was instantiated in a familiar concrete domain (such as legal drinking ages), success rates rose to 70% or more (Johnson-Laird et al., 1972; Griggs and Cox, 1982). Similarly, when LeFevre and Dixon (1986) gave participants a task in which the verbal instructions and the concrete instructional example were inconsistent with one another, over 90% of the participants simply followed the example while disregarding the instructions. This suggests a strong preference for the processing of concrete, instantiated information (also see Anderson et al., 1984; Ross, 1987). In fact, meaningful connections to existing knowledge seem to play a fundamental role in memory and understanding. For example, Bransford and Johnson (1972) found that recall and rated comprehension for a written passage doubled when it was framed in terms of a familiar schema. It is therefore no surprise that meaningful, concrete examples are encouraged in pedagogy—they are typically associated with substantial improvements in comprehension, memory, and reasoning.

### Cognitive Costs of Concreteness

Despite these compelling benefits, more recent evidence suggests that concrete instructional examples may also come with some significant costs. For example, any extraneous detail in the presentation of information tends to distract learners from the relevant content, leading to poorer recall for that material (the “seductive details effect”; Garner et al., 1989; Harp and Mayer, 1998). More insidiously, even those concrete details that are integral and relevant to the examples may harm learning by impairing transfer to new situations. For example, Goldstone and Sakamoto (2003) taught participants about the principle of *competitive specialization* through a simulation portraying ants foraging for food. In this case, neither the ants nor the food were superfluous to the learning—they were integral components of the training example. The researchers nonetheless found that the perceptually detailed representation of these entities could impair participants’ ability to generalize their knowledge to a new, dissimilar case, especially for poorer learners. When the ants and food were depicted in a more abstract, idealized form (as dots and color patches), transfer was improved. Other studies using very different kinds of materials have reported similar patterns (e.g., Clement et al., 1994; Kaminski et al., 2008). In all of these cases, the concreteness that likely improved learners’ comprehension also appeared to more firmly embed the concepts in their original specific context. When confronted with a new analogous situation in which the surface details were changed, the richness of the previously learned representations seems to have made it more difficult for individuals to perceive the relevant connections.

In addition to the more objective aspects of concreteness, similar effects may be observed when interacting with materials that are particularly personally engaging. For instance, Son and Goldstone (2009) taught participants the fundamentals of Signal Detection Theory through the example of a doctor diagnosing patients. When the training case was “personalized” by using passages written in the second-person (“Imagine that you are a doctor who looks at blood samples…”), performance on a transfer test was impaired relative to a more general third-person description (“Imagine a doctor who looks at blood samples…”). Even a third-person description, if given a personalized referent (“Juan has to know how many gallons of milk…”), can lead to poorer transfer than non-personalized materials (Riggs et al., 2015). DeLoache has found similar patterns in work with younger children (e.g., DeLoache, 1991, 1995; DeLoache and Burns, 1994). In addition to demonstrating poorer generalization from materials that are objectively more concrete, she also finds that personal interaction with the physical training materials can impair transfer. There are competing results that indicate benefits of personalizing learning materials to the goals of the learner (Moreno and Mayer, 2004) and incorporating personal pronouns (“your lung”) vs. a more generic framing (“the lung”; Mayer et al., 2004), but the tests of learning in these cases did not involve generalization to new and dissimilar situations.

Perversely, then, it is those very same qualities that are so beneficial in the learning of new material—concreteness, familiarity, personal relevance—that appear so detrimental to the *generalization* of that knowledge. This is no minor annoyance. The primary goal of education is to prepare students to think *outside* of the classroom. Students may be learning about basic algebra in terms of apples and oranges, but the hope is that they will then be able to appropriately apply these principles in order to understand their mortgages, modify their recipes and calculate their gas mileage. A failure to support generalization could reasonably be interpreted as a failure of education overall (see Day and Goldstone, 2012).

### Integrating Concrete and Idealized Materials

Of course, the relationship between knowledge acquisition and knowledge transfer is more complex than a simple “either/or” dichotomy. Most obviously, acquisition is a *prerequisite* for transfer: students cannot generalize information that they have not first learned. A teacher therefore cannot simply decide to emphasize generalizability while disregarding comprehensibility. The situation is further complicated by the fact that contextualization and concreteness can have effects that vary under different circumstances and with different measures. For instance, McNeil et al. (2009) asked participants to solve money-based word problems either with or without the use of perceptually rich manipulatives (bills and coins). On the one hand, they found that these participants were less successful in generating the correct answer when concrete objects were used, consistent with the previously discussed research. On the other hand, use of these manipulatives was associated with fewer *conceptual* errors. That is, while these participants performed more poorly in their calculations, they appeared to have a more accurate and meaningful understanding of *how* the numbers in the problems should be integrated. Koedinger et al. (2008) also reported a more complex relationship between problem solving and grounded representations, finding that simple mathematical word problems were solved more successfully when they were described verbally, while more complex problems benefited from formal mathematical representations that stripped away the grounded references.

While laboratory studies seem to support a decreased reliance on contextualized materials, one might reasonably be skeptical about whether these results would extend to real students in real classroom contexts. Considering the obvious ramifications of these laboratory results for the field of education, it is particularly important to test these effects in naturalistic educational settings, as the experimental contexts common in laboratory research may not necessarily warrant generalization to authentic classroom environments (Bronfenbrenner, 1976; Newman and Cole, 2004). For one, students enrolled in real classes endure real consequences (namely, worse grades) when they fail to learn the material, and they may have more invested in learning transferable representations than study participants. Moreover, authentic learning contexts would include self-guided study (with frequent opportunities for interruption and distraction), and extended time (days or weeks) between initial exposure and testing. Given the even greater likelihood of distraction and poor comprehension in these environments, it is possible that the balance of priorities would shift in favor of those factors that most directly support student attention and engagement. Consistent with this possibility, some prior work has reported reasonably strong transfer effects from fairly concrete training materials (e.g., Fong et al., 1986).

In order to assess these issues, we conducted two studies involving the presentation of curriculum-based materials in the course of regular instruction. In particular, we sought to answer the questions: In natural educational settings in which students are learning material of direct relevance to their classes, will the use of contextualized materials impair students’ ability to transfer their knowledge to new situations, consistent with most previous laboratory studies? Or, in contrast, will the benefits of improved attention, engagement, and comprehension associated with contextualization prove more important given these learners’ particular vulnerabilities to distraction?

Given that this is an initial attempt to generalize a diverse body of laboratory findings to a more natural environment, we have chosen to construe “contextualization” in a fairly broad manner. For the present purposes, we define contextualization as the inclusion of potentially salient content that embeds the learning material in a specific domain or scenario, but that is extraneous to the general principle of interest. This definition includes increases in the concreteness, or the degree of physical, perceptual detail presented in training materials, as well as superfluous situational information that is not inherently perceptual. Furthermore, we include in our construal the possibility of extraneous detail that is introduced by the learners themselves as a function of their prior knowledge about and associations with a given context. For instance, a dog expert would be likely to experience more contextual effects from an example involving dogs than from one that describes a less personally familiar domain, because the former could engage a large body of existing specific knowledge.

The present experiments manipulate context in a variety of complementary ways. While we make some efforts in our experimental design to distinguish these and assess their relative contributions, in other instances we explore conditions in which a number of different kinds of contextualization are simultaneously brought to bear.

## Experiment 1

Our first study was conducted in undergraduate Introductory Psychology classes. This course can present particular challenges to instructors. The material covered is quite broad, class sizes tend to be especially large, and the typical student is close to the beginning of his or her college career. However, despite the course’s breadth of content, it is traditional for instructors to emphasize experimental research methods, and this material is usually presented early in the course to enable students to understand and interpret the empirical studies that form the basis for the remaining class (Homa et al., 2013).

Our topic of interest was the measurement of central tendency (including mean, median, and mode), which brings a number advantages. First, it is broadly relevant to the course and tends to be covered early across different instructors and textbooks, which allowed us to coordinate participants from several course sections. This topic also involves understanding at different levels of explanation. For example, these measures can be described in a purely procedural way (e.g., the mean is calculated as the sum of the values divided by the number of values) or at a more conceptual level (e.g., the mean is the measurement most influenced by outliers). Finally, the measurements are inherently general and broadly applicable across content domains, allowing us to freely vary the degree of contextualization in the training materials and measure transfer of these concepts to new content areas.

In this study, contextualization was independently varied in two ways: the graphics presented in the training could be rich and realistic or abstract, and the written description could include an engaging backstory or could be sparse and detached. These training materials were presented in either of two content domains; some students’ training involved summarizing exam scores on a math test (a relatively familiar content domain for undergraduates), while others’ involved summarizing the number of quality control tests passed by table lamps. Students were assigned to access the training materials for homework credit, and the final assessment of learning involved test items on standard in-class mid-term exams that were separated from the training by at least 2 weeks.

### Participants

The instructors of six different sections of Introductory Psychology (P101) volunteered to integrate the training materials about measures of central tendency (and corresponding exam questions) into their courses during the Fall 2012 term. Total enrollment across all six sections was 1,447 students. The rights of student participants were protected under a research protocol approved by the Indiana University Institutional Review Board. Participants provided written informed consent during the first week of the semester, and 205 students were excluded from our analysis because they did not provide (or were not present to provide) written informed consent. Another 176 students were excluded because they did not return pretest responses, did not take the subsequent exam, or did not access the tutorial. After these exclusions, 1,066 students remained in our analysis, 639 females and 427 males by self-report. In addition to the ecological validity provided by this group of participants, they also represent quite a large sample size for experimental work of this kind. This allows us to overcome many common inferential problems associated with small samples when interpreting our data.

### Test Questions

We wrote four multiple-choice test questions evaluating P101 students’ knowledge about measures of central tendency (mean, median, and mode). Two of these questions pertained to the mean, median, or mode for exam scores on an elementary school’s math test, and the other two questions involved quality control scores for a set of table lamps. Variants of these same four questions were asked during pretest and posttest, with different datasets to summarize during pretest and posttest, different wordings, and different answers and answer orders. Two of the questions, one for each domain, involved calculating median, mode, and mean scores. The other two questions were conceptual, requiring students to understand how the distribution of scores differentially affects median, mode, and mean. For example, a conceptual lamp question was: “If most lamps pass about the same number of tests, but one lamp passes far fewer tests, will this lamp’s performance affect the mode or the mean more?” (All materials and datasets are available online through Open Science Framework at the following link: osf.io/kdf6y/).

### Training Materials

**FIGURE 1:**
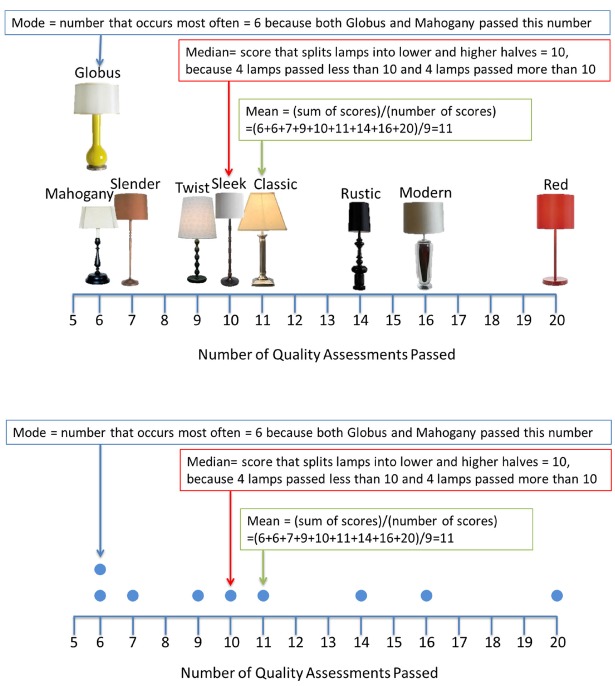
**Detailed (top) and idealized (bottom) graphics from the training in Experiment 1**.

Training consisted of a set of instructions about calculating means, medians, and modes, followed by a series of nine questions (with response-level feedback), implemented online using Qualtrics. The questions all involved students’ calculating means, modes, or medians for a given data set. Accordingly, the training was more procedurally oriented than the conceptual test questions. There were three different manipulations of this tutorial: (1: *Domain*) the instructions and training questions were either about summarizing grades on a math test (“grades” domain) or about summarizing quality control tests for a set of table lamps (“lamps” domain); (2: *Graphics*) the instructions either included real pictures of students and table lamps (“detailed” graphics), or dots to symbolize these students and lamps along a number line (“idealized” graphics; similar to Kaminski and Sloutsky, 2013); and (3: *Immersion*) the instructions either included an immersive backstory describing the situational importance of calculating these measures of central tendency (e.g., a fight has broken out over the correct way to summarize math scores; “high” immersion) or a simple statement that it is sometimes useful to have different summary statistics (“low” immersion). Furthermore, the high immersion materials were written in the second-person (“Imagine that you are invited into a classroom as an expert statistician”) while those for the low immersion group were in a more neutral third-person. These three manipulations were fully crossed (domain × graphics × immersion; 2 × 2 × 2) for eight different versions of the tutorial (see Figure 1 for sample materials).

### Procedure

We visited the six P101 classes in person during the first week of the semester, and invited all students to take a short ungraded multiple-choice pretest and to consent to our analysis of their P101 coursework. Even though the pretest was ungraded, students were encouraged to pay attention to the questions because very similar questions would appear on their next test. During the second week, when students were learning about psychology research methods, instructors assigned them to do the training tutorial for homework credit, to be completed prior to the subsequent test date. Every student was randomly assigned to one of the eight versions of the tutorial, and these tutorials were linked from class websites (access was restricted so that students could only see the link for their assigned version of the tutorial). On the subsequent exam (either the third or fourth week of the semester, depending on the section), the four post-test questions about central tendency were embedded among the other P101 multiple-choice test questions, presented sequentially in the same order as in the pretest.

### Results

A mixed design ANOVA was conducted, including two within-subjects factors (test item domain: math grades vs. lamps, and test item type: conceptual vs. calculation) and four between-subjects factors (training domain: lamps (low familiarity) vs. math grades (high familiarity), graphics: detailed vs. idealized, story immersiveness: high vs. low, and P101 section), with improvement between pretest and posttest as the dependent variable. (see Table 1, for a summary of results.)

**TABLE 1:** **Summary of results from Experiment 1**.

	**Intra-domain (trained)**			**Extra-domain (transfer)**		
**Factor**	**Pretest**	**Posttest**	**Improvement**	**Pretest**	**Posttest**	**Improvement**
Immersion*
High	1.44	1.77	0.33 (0.75)	1.44	1.76	0.31 (0.71)
Low	1.48	1.78	0.30 (0.75)	1.42	1.82	0.40 (0.76)
***Graphics***
Detailed	1.45	1.77	0.33 (0.77)	1.44	1.78	0.33 (0.74)
Idealized	1.47	1.78	0.30 (0.73)	1.42	1.80	0.39 (0.73)
Training domain**
Lamps	1.45	1.66	0.21 (0.81)	1.40	1.89	0.49 (0.68)
Grades	1.47	1.89	0.42 (0.67)	1.46	1.68	0.23 (0.77)
Overall	1.46	1.77	0.32 (0.75)	1.43	1.79	0.36 (0.74)

*Indicates p < 0.05; ** indicates p < 0.001.

**FIGURE 2:**
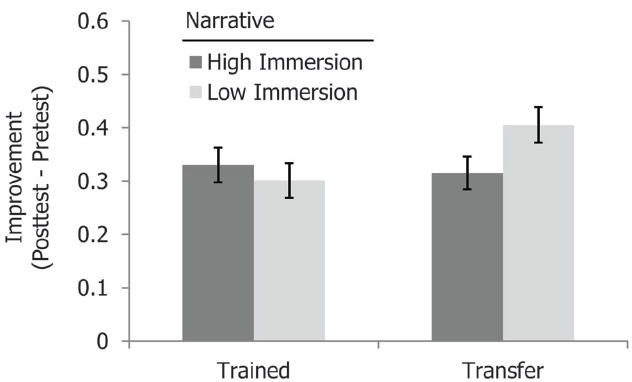
**Mean posttest improvement in Experiment 1.** Transfer was superior when training involved a less immersive narrative. Error bars represent standard errors.

Our primary interest in this study was in the effects of contextualization on transfer to new domains. To explore such effects, we examined the interactions between the training and test domain, and particularly how these were influenced by the varied contextual factors. A three-way interaction between training domain, test domain and immersiveness [*F*(1,1018) = 4.09, MSE = 0.876, *p* = 0.04, ${\text{η}}_{\text{p}}^{\text{2}}$ = 0.004] showed that transfer was indeed influenced by the contextual detail of the story (see Figure 2). Specifically, transfer to a new domain (measured by the test items that were from a different domain from a participant’s training) was most successful when the training material was less immersive, rather than when it involved the relatively engaging back story [*M* = 0.40 (SD = 0.76) vs. 0.31 (SD = 0.71), *F*(1,1065) = 3.96, MSE = 2.15, *p* = 0.047, ${\text{η}}_{\text{p}}^{\text{2}}$ = 0.004]. No such difference was found for non-transfer test items [items that matched the participant’s training domain; *M* = 0.30 (SD = 0.75) vs. 0.33 (SD = 0.75)]. A numerically similar pattern was found for graphical concreteness, with greater transfer after training with materials that were *less* graphically detailed [*M* = 0.39 (SD = 0.73) vs. 0.33 (SD = 0.75)], although this difference did not reach statistical significance, perhaps because the graphics were not as captivating as the immersive backstory. Overall, then, our data are consistent with previous research showing that knowledge transfer is best supported by less contextualized learning. There was a significant main effect of P101 section [*F*(5,1018) = 2.88, MSE = 0.916, *p* = 0.014, ${\text{η}}_{\text{p}}^{\text{2}}$ = 0.01], as students in different classes had different improvements from pretest to posttest. However, there were no significant interaction effects between P101 section and any of the experimental factors manipulated in this study.

An additional focus of interest in this study was in the role of domain familiarity in these effects. A main effect of test domain [*F*(1,1018) = 70.48, MSE = 15.12, *p* < 0.001, ${\text{η}}_{\text{p}}^{\text{2}}$ = 0.07] reflected the fact that items involving math grades improved substantially more than items involving lamp ratings [*M* = 0.46 (SD = 0.68) vs. *M* = 0.22 (SD = 0.79)]. This effect held regardless of training domain, and was entirely the result of differences in post-test scores, with pre-test performance being very similar for the two types [*M* = 1.43 (SD = 0.62) vs. 1.45 (SD = 0.70; out of a possible 2)]. Overall, participants in this study had an easier time applying their learned knowledge to a more familiar domain. Interestingly, this was true even when students’ initial training was in the less familiar domain of lamp ratings.

**FIGURE 3:**
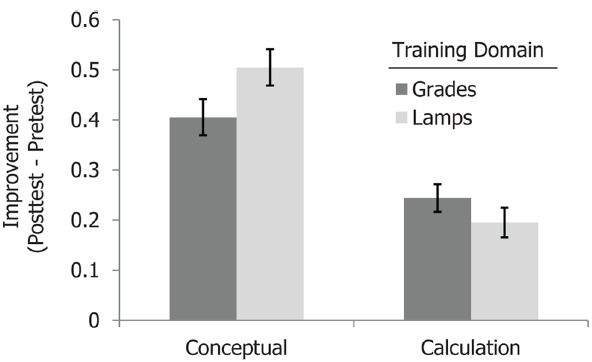
**Means showing the interaction between training domain and test item type in Experiment 1.** Training in the less familiar domain led to greater improvement on conceptual test items. Error bars represent standard errors.

Analysis also revealed a relationship between domain familiarity and the type of test item (procedural vs. conceptual). Specifically, an interaction between item type and training domain [*F*(1,1018) = 6.82, MSE = 1.648, *p* = 0.01, ${\text{η}}_{\text{p}}^{\text{2}}$ = 0.007] reflected the fact that there was greater improvement on the conceptual items after training in the *less* familiar domain [lamp quality scores; conceptual improvement *M* = 0.50 (SD = 0.84)] vs. the more familiar domain of grades [conceptual improvement *M* = 0.41 (SD = 0.83); see Figure 3]. One possible interpretation of this result is as an example of “desirable difficulty” (Bjork, 1994). That is, students who were trained with materials from an unfamiliar domain were less able to rely on their existing knowledge structures to scaffold their understanding. While this would have made initial comprehension more challenging, this difficulty would have demanded that students process the material more deeply in order to achieve an appropriate level of understanding, leading to a stronger conceptual grasp of the material. The unfamiliar domain would have posed less of a problem for (and therefore provided less of a benefit for) the more procedural calculations of central tendencies. Consistent with this interpretation, we did not observe a similar advantage for these [*M* = 0.24 (SD = 0.64) vs. 0.20 (SD = 0.68), for grades and lamp ratings, respectively].

### Discussion

Contextualization in learning can involve important potential tradeoffs between the ease of understanding material and the ease of generalizing it. While a growing body of research has found advantages for decreasingly concrete training materials in situations where transfer is the ultimate goal, there was reason to question whether this effect would hold in actual educational environments, where the high potential for distraction might give a greater priority to the facilitation of comprehension afforded by concreteness. Our results did not support this possibility, however. Consistent with previous research, students in our study were better able to apply their learned knowledge to a new domain when it had originally been presented during training without an engaging narrative context. Thus, even in an introductory undergraduate educational context in which attention and engagement may be challenging to maintain, the net costs of contextualization appeared to outweigh any potential benefits.

We also discovered significant effects of the familiarity of the content domain. Overall, students had an easier time learning within the familiar domain of grades, and test items on this subject showed significantly greater improvement than items from the less familiar domain. On the other hand, we found evidence that *training* in a less familiar area could lead to a better conceptual understanding of relevant principles. When students were trained in the unfamiliar domain of lamp quality ratings, they showed greater overall improvement on the conceptually-oriented test questions.

These results raise interesting issues regarding the relationship between familiarity and contextualization. In one sense, familiarity represents a kind of additional context, because it suggests that individuals will be supplementing any given material with a large body of previously known facts and details. This interpretation is consistent with some of the previously discussed literature in which greater personal relevance was associated with decreased transfer (e.g., Son and Goldstone, 2009), and it helps to explain the enhanced conceptual understanding that we found following training in an unfamiliar domain. At the same time, the processing benefits associated with this familiarity seemed to provide important advantages in those situations that did *not* involve the acquisition of new conceptual knowledge. Students’ improvement was greatest on test items from the familiar domain of grades, and training in that domain was statistically equally good at supporting transfer of simple procedural content. Familiarity, like other more concrete kinds of contextualization, seems to be associated with both important costs and benefits.

## Experiment 2

The first experiment provides evidence that in educational contexts, as in prior laboratory research, concrete detail may be detrimental to knowledge transfer. The second experiment serves to replicate these results in a younger classroom population and extend them to cases of far transfer.

While the overall research goals of this study are similar to those of Experiment 1, we modified the operationalization in several ways. First, the participants in this experiment were seventh and eighth grade students rather than college undergraduates. Second, the learning phase of the study involved an in-class activity rather than a self-guided homework assignment. We also attempted to extend the manipulation of contextualization outside of the actual learned example. Whereas the materials in Experiment 1 varied the concreteness, immersiveness and familiarity of the training example itself, the example in Experiment 2 is identical for all participants. The only factor that varies between individuals is the immersiveness of a separate paragraph preceding the training task that serves to motivate interest in the topic, although it is not directly relevant to understanding it. Moreover, these immersive paragraphs were written without reference to any specific person, allowing us to explore the effects of immersion without the potential confounds of personalization.

The study also makes use of a different learning topic. Positive feedback systems, in which changes to one part of a system produce effects that lead to additional changes to that initial part, are widespread in any number of domains, and are particularly relevant in the sciences. (For simplicity with our population, we described positive feedback in terms of mutual influence between two parts of a system, e.g., A increases B, and B further increases A.) The training materials in this study involved processes of positive feedback in the reflectance (or *albedo*) of the Earth’s polar ice, an important factor in global climate change. Finally, Experiment 2 made use of a different dependent measure of conceptual knowledge: students’ ability to classify new cases according to their underlying structure. Classification is a fundamental cognitive process, and it serves as a basis for many other processes. Among other things, it is particularly relevant within complex problem solving. For example, individuals with expertise in physics are able to classify problems according to their meaningful structural commonalities (e.g., problems based on conservation of energy) rather than their more immediately obvious surface characteristics (e.g., problems involving an inclined plane; Chi et al., 1981). Because of this, these experts will be much more likely than novices to apply appropriate problem strategies and generate accurate solutions.

Our use of a middle school population also allowed us to examine possible individual differences in the effects of context. A significant minority of our students were participating in an accelerated science program within the school, reflecting achievement on a test of scientific knowledge. There are many independent factors that could contribute to membership in this program, including innate ability, high need for achievement, and interest in the subject matter. However, these students tend to possess a relatively deep understanding of the material. Prior research has shown that expertise in a field is associated with a greater ability to focus on the relevant structural content of a problem while being less influenced by any irrelevant “surface” features (e.g., Chi et al., 1981; Schoenfeld and Herrmann, 1982). Because of this, it is possible that students in the accelerated program will prove less susceptible to the influence of irrelevant contextual information in our study. This would be consistent with the results of Goldstone and Sakamoto (2003), who found that perceptual concreteness exerted the most influence over those who performed poorly on the initial task.

Given that participants in our task were asked to determine whether new cases follow a particular underlying structure, we were also able to assess the possibility of bias effects. That is, it is possible that posttest responses would be biased toward classifying all of the examples as instances of positive feedback—the principle underlying their training simulation. Our primary questions will be whether any such bias effects might interact with the contextualization of the materials.

### Participants

This study was conducted with 144 students at a public middle school, who participated as part of their regular class time in a General Science course. These included 70 seventh-grade students and 74 eighth-grade students, drawn from six different class periods meeting throughout the day. Participants were roughly evenly divided between males (*n* = 68) and females (*n* = 74), and approximately one-third of these students (*n* = 49) were part of the school’s accelerated learning programs (ALPs), composed of individuals who had passed a rigorous science achievement test. Eleven students were dropped from the final analysis because of failure to complete the test materials. The rights of all student participants were protected under a research protocol approved by the Indiana University Institutional Review Board. Written consent was obtained from the parents of all students at the beginning of the semester, and was separately obtained from the students themselves at the beginning of the class session.

### Materials and Design

Our measure in this experiment was students’ ability to correctly identify scenarios as examples of positive feedback systems, tested both before and after training. Training itself involved a written description motivating the topic, followed by an interactive computer simulation demonstrating the phenomenon of positive feedback in a specific concrete context: global climate change. Testing and training were identical for all students, with the exception of a brief motivating paragraph at the beginning of the written instructions, which varied between participants in terms of its narrative richness and immersiveness.

The test materials were designed to assess students’ understanding of positive feedback systems across a wide range of domains. First, students read a definition of positive feedback (which was slightly simplified in order to be understandable by their age group; see online materials). Students then read eight brief scenarios describing a variety of real-world phenomenon, half of which were examples of positive feedback systems. For each scenario, students were asked to rate whether the described situation was an example of positive feedback on a five-point scale ranging from *Definitely not* to *Definitely yes*.

### Instructions and Simulation

**FIGURE 4:**
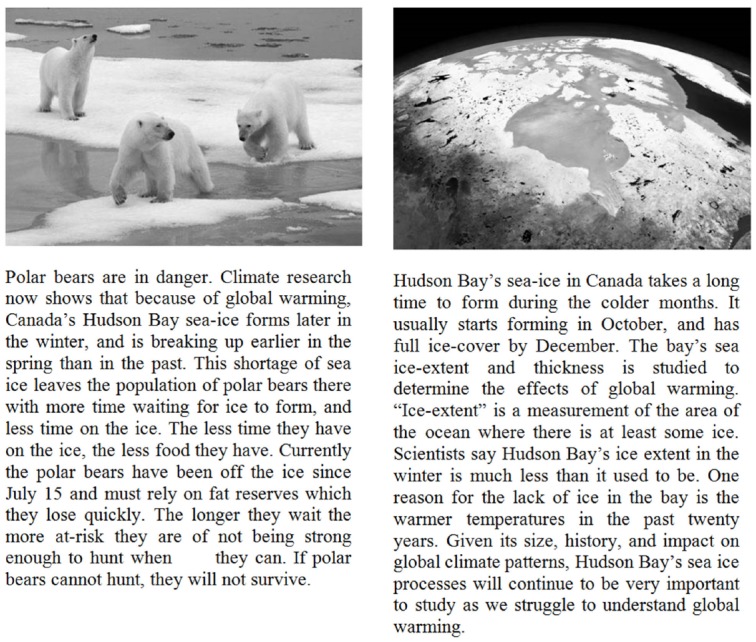
**High (left) and low (right) context materials for Experiment 2.** These materials were shown prior to the description and instructions for the training task.

All students read introductory materials (presented on computer) describing *ice-albedo feedback*, a kind of interaction between the reflectivity of polar ice and the absorption of solar heat. The first page of these instructions was a brief paragraph motivating the topic by describing the impact of global warming. For roughly half of the students (the *Low Context* group, *n* = 75), this paragraph described recent patterns of melting in the Hudson Bay area (see Figure 4). This topic is relevant to the issue of global climate change and is straightforward to understand. However, for most individuals in our study it is not particularly personally relevant or emotionally engaging, and does not draw on much existing background knowledge. The remainder of the students (the *High Context* group, *n* = 69) read a motivating paragraph describing the effects of climate change on polar bear populations. This passage was designed to be more directly relevant to students’ knowledge and interests, and it described the plight of these animals in more personal terms.

This paragraph was followed by a general introduction to the task and a description of ice-albedo feedback effects. All students then interacted with a computer simulation of ice-albedo feedback (developed and implemented in NetLogo; Wilensky, 1999). Students were guided through the task with specific instructions designed to highlight the feedback behavior of the system.

**FIGURE 5:**
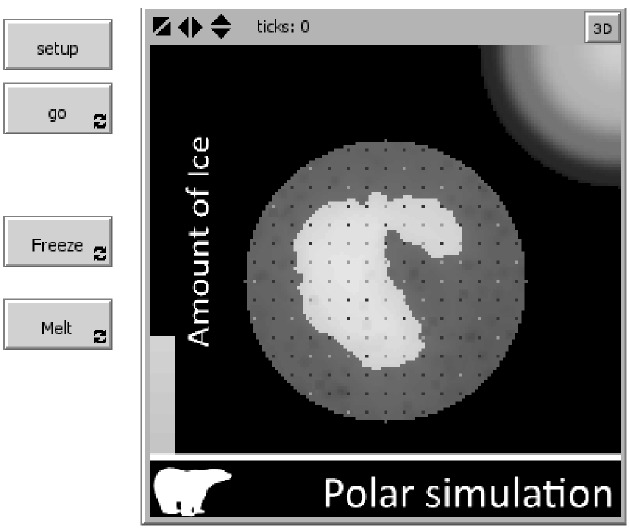
**Ice-albedo simulation used in Experiment 2**.

The simulation itself displays a top-down view of a polar ice cap, surrounded by water (see Figure 5). The primary dynamics of the system involve the size and shape of the ice surface, which is constantly changing. These changes are driven by several factors, including general cooling and diffusion of heat. The most relevant factors for the students, however, are sunlight and albedo. The earth is receiving a steady flow of energy in the form of sunlight, which can be absorbed and can increase an area’s temperature. However, not all of this energy is absorbed: much of the light is reflected back into space. Furthermore, the amount of light that is reflected depends on a given area’s albedo or reflectance. Critically, ice has a much higher albedo than land or water, because of its white color. In our simulation, ice only absorbs one quarter of the energy that is absorbed by the surrounding water. Because of this, greater ice coverage results in less overall warming. It is this factor that produces the system’s feedback behavior. A decrease in ice coverage results in more heat being absorbed, leading to even more melting, and so on. Conversely, an increase in ice coverage causes the reflection of more light, reducing the temperature and potentially causing even more water to freeze.

The simulation uses a grid of colored points to indicate each region’s overall light reflectance and absorption (the “reflection grid”). Red dots (darker in Figure 5) indicate absorbed energy, while blue dots (lighter) indicate reflected energy. This grid provides a way for students to directly perceive the relative balance of reflected and absorbed energy in different locations. Specifically, 80% of the dots on frozen areas show reflectance (blue dots), compared with 20% of the dots on the water. When active, this grid flashes on and off in one second increments.

Students were guided through the simulation via specific instructions, given through popup messages. Initially, students were familiarized with the operation of the system, first without the reflection grid, and then with. Messages appeared at brief intervals reminding them of the relevant principles of the system (see online materials for full protocol). Next, students were instructed to interact with the system in various ways. For example, students used the simulation’s controls to melt a significant area of the ice. At this point, the reduced albedo led to a positive feedback loop in which additional ice melted at an accelerating rate, until eventually all of the ice had melted. Next, students used the controls to observe the complementary feedback effect, with greater ice coverage causing additional freezing. After each of these tasks, students were explicitly reminded of the way in which this reflected positive feedback behavior.

Finally, students were able to freely interact with simulation for up to 3 min. Additionally, at this point we added sliders that allowed students to directly control the reflectance of ice and water in the simulation.

After the simulation, students were asked to define positive feedback systems in their own words: “We would like you to tell us what a positive feedback system is. Just do your best to describe it in your own words. Please don’t just write about the simulation you just saw. Instead, try to write about positive feedback systems in general.”

### Results

We coded the pre- and post-training classification tests according to each response’s proximity to the correct end of the rating scale. For instance, if a particular scenario described a positive feedback system, then a response of “Definitely yes” would be assigned a score of four and a response of “Definitely not” would be assigned a score of 0, with intermediate responses receiving appropriate intermediate scores. For scenarios that were not examples of positive feedback this coding would be reversed, with “Definitely not” receiving a score of 4 and “Definitely yes” receiving a score of 0. Each participant was assigned an improvement score calculated as the difference between totaled pretest and posttest scores.

A 2 (condition) × 6 (class period) ANOVA revealed a significant effect of condition on posttest improvement [*F*(1,121) = 4.36, MSE = 20.71, *p* = 0.039, ${\text{η}}_{\text{p}}^{\text{2}}$ = 0.035], but no effect of class period [*F*(5,127) = 1.45, n.s.], and no interaction between condition and class period [*F*(5,127) = 1.24, n.s.]. Specifically, planned comparisons showed that those students in the Low Context condition improved significantly between pretest and posttest [*M* = 1.58 (SD = 3.97), *F*(1,68) = 10.91, MSE = 7.89, *p* = 0.002, ${\text{η}}_{\text{p}}^{\text{2}}$ = 0.138] while those in the High Context group showed a small numerical decrease in performance after training [*M* = –0.36 (SD = 5.21), *F*(1,63) = 0.31, n.s.]. The decrease in posttest performance by the High Context condition meant that no significant improvement was seen when pooling across all students [*M* = 0.65 (SD = 4.69), *F*(1,132) = 2.53, MSE = 11.00, *p* = 0.114, ${\text{η}}_{\text{p}}^{\text{2}}$ = 0.019].

To ensure that this effect was not being driven by a small subset of the test items, we also performed an item analysis. A repeated measures *t*-test, comparing each item’s average posttest improvement in Low [*M* = 0.20 (SD = 0.25)] and High [*M* = –0.04 (SD = 0.31)] context groups, confirmed a difference between conditions [*F*(1,7) = 6.10, MSE = 0.039, *p* = 0.043, ${\text{η}}_{\text{p}}^{\text{2}}$ = 0.465]. For seven of the eight test items, posttest improvement was greater under conditions of low contextualization.

One possible explanation for the poorer performance by the High Context group is that those students were confused or distracted by the additional engaging information. If so, we should expect that group to have a poorer understanding of the material that was learned in training, in addition to their decreased scores on the classification task. To assess this possibility, we used a five-point rubric to code the accuracy of students’ posttest attempts to define positive feedback. In simplified terms, this rubric was as follows: (1) basic statement of cause and effect; (2) statement that a change in one variable causes a change in another; (3) inclusion of direction of change; (4) inclusion of mutual influence (second variable influences the first); (5) mention of continuing loop and consequences (e.g., movement toward a maximum or minimum). We found no difference between the groups on this measure, with those in the High Context condition actually showing a slight numerical advantage over the Low Context group [*M* = 2.11 (SD = 1.53) and 1.99 (SD = 1.46), respectively, out of a possible 5; *F*(1,132) = 0.48, n.s.]. The difference in classification performance therefore cannot be attributed to poorer initial learning, but instead seems to reflect a greater difficulty in applying the knowledge acquired during interaction with the simulation to new situations.

**FIGURE 6:**
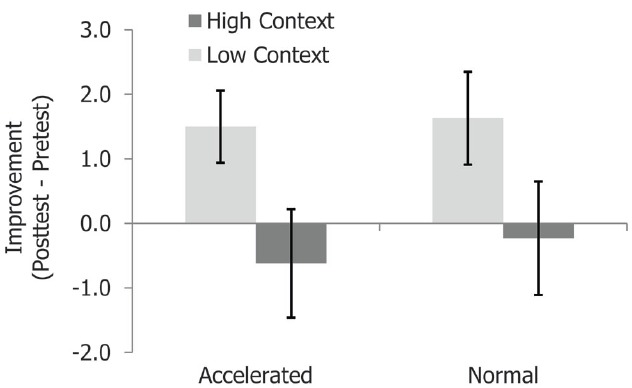
**Means from Experiment 2 for accelerated and non-accelerated students.** Performance in both groups was equally affected by richer background context. Error bars represent standard errors.

Next, we compared the performance of students in the accelerated ALPs program with that of their (non-accelerated) classmates. We had hypothesized that these potentially more advanced or engaged students might be better able to focus on the relevant structural content of the training, and would therefore show a smaller effect of contextualization. However, this was not the case. A 2 (ALPs membership) × 2 (condition) ANOVA on improvement scores revealed no main effects for accelerated class membership [*F*(1,132) = 0.10, n.s.] and no interaction between ALPs membership and condition [*F*(1,132) = 0.02, n.s.; see Figure 6]. Thus, while those in the ALPs group outperformed their peers at both pretest [*F*(1,131) = 48.84, MSE = 24.65, *p* < 0.001, ${\text{η}}_{\text{p}}^{\text{2}}$ = 0.272] and posttest [*F*(1,131) = 41.36, MSE = 28.32, *p* < 0.001, ${\text{η}}_{\text{p}}^{\text{2}}$ = 0.24], their overall improvement and susceptibility to context effects did not differ from that of their classmates. Like the non-accelerated students, performance for the ALPs students in the High Context condition actually decreased numerically relative to pretest [*M* = –0.62 (SD = 3.85)].

Finally, we examined the possibility of bias effects in students’ responses. To measure whether students were biased to make responses that were consistent with the subject of their training, we calculated each response’s proximity to the end of the rating scale marked “Definitely yes,” regardless of whether this was the correct response. Therefore, each “Definitely yes” response was scored as a four, each “Definitely not” response was scored as a 0, and intermediate responses were assigned the appropriate intermediate value. For each participant, bias was coded as the difference in this score between pretest and posttest.

Across all students, this average bias score was significantly greater than 0 [*M* = 0.30 (SD = 1.01), *t*(132) = 3.47, *p* < 0.001, *d* = 0.30]. Overall, students were more likely to classify a scenario as an example of positive feedback after having received training, regardless of the accuracy of that classification. However, a 2 (ALPs membership) × 2 (condition) ANOVA on the scores showed that this bias effect did not vary as a factor of condition [*F*(1,132) = 0.32, n.s.] or ALPs membership [*F*(1,132) = 1.04, n.s.].

### Discussion

As in Experiment 1, students in this study were negatively affected by exposure to a richer, more engaging background story for the learning example. High Context students showed absolutely no posttest improvement as a result of their training. These effects were found despite the use of a relatively subtle manipulation. All of the students in this experiment received identical training and instructions, with the only difference between conditions being the immersive vs. detached nature of a motivating paragraph prior to the training simulations that included content only indirectly related to the simulation itself.

Furthermore, we found the effects of a rich context to be resistant to learners’ prior knowledge. Prior research has shown that individuals with greater expertise in a subject area are better able to disregard irrelevant surface details and focus instead on the underlying structure of a situation (e.g., Chi et al., 1981; Novick, 1988). Students in the accelerated science program could reasonably be expected to have greater expertise in general scientific principles than their non-accelerated peers, and consistent with this their pretest and posttest performance was significantly superior to that of their classmates. Despite this, we found them to be equally vulnerable to our context manipulation.

However, these effects did not appear to be the result of a poorer understanding of the relevant principles by those receiving a richer context. When students were asked to define a positive feedback system in general terms at the end of the experimental session, those who had read the more engaging motivating paragraph produced an equally accurate definition as those who had read the more detached passage.

If poorer understanding is not the cause, then what is the source of these students’ impaired transfer? We would argue that the rich context provided by the High Context passage served to tie students’ understanding of positive feedback systems more tightly to this one particular content area. Rather than being able to represent the principles in a more contextually independent and general way, through concepts such as variability, causation and mutual influence, their understanding was intimately tied to a single concrete example involving heat and reflectivity.

On reflection, one interesting way in which the contextualization may have occurred in this case is that the inclusion of the polar bear may have drawn participants’ attention to the specifically *negative* change in this feedback example (the decreasing ice habitat). Since all of the correct posttest items involved *positive* change, this more specific representation may have put the High Context group at a particular disadvantage. Thus, the concrete contextualization in this example may have influenced participants’ representations of positive feedback at a fairly deep and abstract level [Schwartz (2015), personal communication].

## General Discussion

There are solid practical reasons for the traditional advice that educators should use concrete, meaningful examples in instruction. Research has consistently shown that this type of contextualization can enhance understanding, improve retention, and facilitate effective reasoning about the subject matter. Furthermore, making connections to students’ existing knowledge and interests can help to maintain their attention and motivation, which are critical factors for learning. However, research has also shown that there are costs associated with this approach: the more contextualized a training case, the more difficulty learners can have in recognizing and applying its principles in new and dissimilar situations.

The undermining of generalization and transfer by contextual detail could be particularly problematic in general education, where the ultimate goal is typically to train students for a wide and unpredictable variety of real-world applications. We had reason to question whether contextualization would actually be as detrimental in the classroom as previous laboratory research has suggested, however. In particular, it would be reasonable to suggest that attention and motivation would be especially difficult to maintain in these environments and populations, and that the benefits associated with engaging and meaningful material might exceed any of the conventional costs. Furthermore, whereas the materials learned in a laboratory experiment may be interpreted as restricted in relevance to that particular and peculiar context, materials learned in a classroom context may be construed in a more expansive manner if taught well (Engle et al., 2012). However, this did not appear to be the case. In two experiments utilizing different populations of students enrolled in science classes, material from different subject areas, different delays for retention, and different types of tasks and tests, we again found that greater contextualization impaired students’ ability to apply their knowledge in new cases.

Interestingly, this was the case even though our studies represented a kind of *cued* rather than *spontaneous* recall. A large body of previous research has established that concrete dissimilarities typically impair individuals’ ability to recognize when prior knowledge is relevant (e.g., Simon and Hayes, 1976; Weisberg et al., 1978; Gick and Holyoak, 1983). In our studies, however, participants were explicitly aware of the relevant concept and example. Contextualization was therefore leading to specific issues with mapping the original case to new examples.

Our results indicate important differences between materials that promote good immediate performance and those that promote transfer. In Experiment 2, definitions of positive feedback loops as they pertained to the training simulation were not better for the Low Context narrative compared to High Context, but transfer was better in the Low Context condition. In both experiments, transfer was best with relatively simple and detached narratives. This is somewhat reminiscent of earlier results showing that understanding of a scientific concept from a simulation was best when simulation elements were relatively detailed and concrete, but that transfer of the concept to a superficially dissimilar simulation was best when the training elements were idealized (Goldstone and Sakamoto, 2003). One implication of this pattern of results is that as an instructor designs their instructional materials, they should be asking themselves whether they are trying to optimize their students’ demonstrated mastery of the material itself or their ability to transfer their understanding to new materials. While we might intuitively believe these to be highly correlated, design decisions about narrative immersion appear to affect these learning outcomes in opposite ways.

What lessons should educators take from these data? To address this question, we must first consider the scope of our results. The present studies represent an attempt to generalize laboratory results to authentic educational contexts. However, in reality they reflect only a limited subset of educational experience. For example, the learning episodes in our experiments involved expository instruction rather than constructive learning exercises such as learning by invention (e.g., Schwartz and Martin, 2004). While there was some degree of interactivity in the computer simulations used in Experiment 2, this is far removed from the degree of flexibility and involvement in an interactive group, where students are free to ask clarifying questions and instructors and peers may adapt their responses in real time. It therefore remains to be seen how broadly these effects may generalize to other instructional settings.

The precise origin of these effects is also unknown. By definition, contextually-rich learning materials, such as those that we’ve manipulated in this study, are multifaceted. They may involve personalization, urgency, social pressures, and a cornucopia of other factors. Future research may ultimately attempt to isolate these effects under ideal comparison conditions, but in the current study, we instead opted to create materials that were more pertinent to routine educational practice, a sort of *pragmatic trial* approach (Roland and Torgerson, 1998). These immersive qualities (the familiarity of the domain, the presence of an alluring backstory, the inclusion of pictures and graphics, etc.) would all commingle in routine classroom settings. While our current work lacks the explanatory power of a laboratory study, it nevertheless demonstrates that the combined effects of contextually-rich training materials are indeed germane to naturalistic learning scenarios.

Even acknowledging these limitations and taking our results at face value, the immediate pedagogical implications of our results are not entirely clear. Should we conclude that meaningful concrete examples should be eliminated from the classroom? There are several reasons why we do not believe that this is the correct conclusion to draw. First, as suggested above, whether narrative concreteness is positive or negative depends on whether one is teaching for immediate performance or future transfer. Second, it is important to remember that all of the materials in our studies were in fact contextualized. There were no control conditions in which information was presented in a completely abstracted or idealized manner. If the concepts involved in central tendency measurements or feedback systems were given without any meaningful context at all, it is likely that students would indeed have had a very difficult time understanding them, and that both learning *and* transfer would have suffered as a consequence. The issues addressed by our data are therefore not so much about the *use* of meaningful examples as they are about the *degree* of context that will be most effective.

Furthermore, the potential benefits of contextualization are substantial enough that researchers have invested a great deal of time into finding ways to overcome their costs (see Day and Goldstone, 2012). Most of these approaches involve the presentation of multiple concrete cases. However, simply being exposed to more than one training example is often not effective on its own—the way in which these cases are chosen and presented has been shown to matter a great deal. For example, Goldstone and Son (2005) found evidence for improved transfer after training following a pattern of “concreteness fading.” In that research, participants first learned about a set of principles through a richly instantiated set of materials. Afterward, this initial concreteness was “faded” from the instruction as participants interacted with materials that presented the same relevant principles in a more idealized way. An even more complete concreteness fading regime that goes first from concrete to idealized, and then from idealized to formal symbolic, representations has been shown to fairly consistently improve generalization of training (Fyfe et al., 2014). Evidently the initial, more contextualized presentation allows learners to scaffold their understanding by connecting it to their existing knowledge and schemas, while the subsequent idealized case helps to dissociate these initial representations from their original context (also see Kotovsky and Gentner, 1996; Scheiter et al., 2010). Thus, learners in this condition seem to gain at least some of the benefits of both concreteness and genericity.

Another method that has been shown to be quite effective in supporting transfer is the active comparison of multiple training cases. For example, Loewenstein et al. (2003) taught a group of management students about the use of “contingency contracts” as a technique in negotiation. All of these students learned about the technique by reading two concrete instantiations of the method in practical application. However, those who simply studied the two cases in succession showed no benefit from the training, and they were no more likely to apply the principles appropriately than a control condition that had received no training at all. Students who had actively compared the two cases and identified commonalities between them, however, were nearly three times as likely to successfully apply their knowledge. Many other studies have found similar benefits of comparison (e.g., Gick and Holyoak, 1983; Catrambone and Holyoak, 1989; Cummins, 1992; Gentner et al., 2009; Christie and Gentner, 2010; Richland and McDonough, 2010).

The question of contextualization in instruction is therefore neither simple nor settled, but we are progressing in our understanding of the issues involved. Simply adding richer meaningful content to in-class examples may make intuitive sense, and may have immediately obvious benefits in terms of student engagement and comprehension. But as the results of our experiments make clear, these short-term benefits seem to come at the cost of students’ long-term ability to apply their knowledge. If educators are to take advantage of these inherent benefits, they will need to give careful consideration to how such examples are designed and used together in order to plan the most effective instruction.

## Author Contributions

SD was responsible for the first drafting of this manuscript, and was involved in all subsequent revisions. SD was also directly involved in the conceptualization, creation of stimuli, materials and interactive simulations, running of participants, and analysis of data for Experiment 2. BM was directly involved in the conceptualization, creation of stimuli and materials, running of participants and data analysis of Experiment 1. He provided additional content and valuable critical revisions to the manuscript. RG was directly involved in the conceptualization of both Experiments, and provided invaluable ongoing contributions and feedback throughout the experimental process. He also provided additional content and valuable critical revisions to the manuscript. All three authors have approved the final version of the manuscript, and agree to be accountable for all aspects of the work.

### Conflict of Interest Statement

The authors declare that the research was conducted in the absence of any commercial or financial relationships that could be construed as a potential conflict of interest.
